# Technical analysis, contestation and politics in policy agenda setting and implementation: the rise and fall of primary care maternal services from Ghana’s capitation policy

**DOI:** 10.1186/s12913-016-1576-2

**Published:** 2016-07-29

**Authors:** Augustina Koduah, Han van Dijk, Irene Akua Agyepong

**Affiliations:** 1 Ministry of Health, P.O.Box MB 44, Ministries, Accra Ghana; 2 Wageningen UR (University & Research centre), Sociology of Development and Change, Wageningen, The Netherlands; 3 Julius Global Health, P.O. Box 85500, 3508 GA Utrecht, The Netherlands; 4 Ghana Health Service, Research and Development Division, Dodowa Health Research Center, Dodowa, Ghana

**Keywords:** Agenda setting, Arenas of conflict, Implementation, Maternal health services, National Health Insurance, Per capita payment

## Abstract

**Background:**

Why issues get on the policy agenda, move into policy formulation and implementation while others drop off in the process is an important field of enquiry to inform public social policy development and implementation. This paper seeks to advance our understanding of health policy agenda setting, formulation and implementation processes in Ghana, a lower middle income country by exploring how and why less than three months into the implementation of a pilot prior to national scale up; primary care maternal services that were part of the basket of services in a primary care per capita national health insurance scheme provider payment system dropped off the agenda.

**Methods:**

We used a case study design to systematically reconstruct the decisions and actions surrounding the rise and fall of primary care maternal health services from the capitation policy. Data was collected from July 2012 and August 2014 through in-depth interviews, observations and document review. The data was analysed drawing on concepts of policy resistance, power and arenas of conflict.

**Results:**

During the agenda setting and policy formulation stages; predominantly technical policy actors within the bureaucratic arena used their expertise and authority for consensus building to get antenatal, normal delivery and postnatal services included in the primary care per capita payment system. Once policy implementation started, policy makers were faced with unanticipated resistance. Service providers, especially the private self-financing used their professional knowledge and skills, access to political and social power and street level bureaucrat power to contest and resist various aspects of the policy and its implementation arrangements – including the inclusion of primary care maternal health services. The context of intense public arena conflicts and controversy in an election year added to the high level political anxiety generated by the contestation. The President and Minister of Health responded and removed antenatal, normal delivery and postnatal care from the per capita package.

**Conclusion:**

The tensions and complicated relationships between technical considerations and politics and bureaucratic versus public arenas of conflict are important influences that can cause items to rise and fall on policy agendas.

## Background

Why some issues get on the policy agenda, move into policy formulation and implementation while others drop off in the process is an important field of enquiry to inform public social policy development and implementation. Despite several published studies on how issues gain prominence [1–4]; or are periodically re-examined and maintained on an agenda over time [5–7] there remains a relative dearth of work on these issues from Low and Middle Income Countries (LMICs) [8].

To advance our understanding in this area of work; the current investigation in Ghana, a lower middle income country explored how in the implementation processes of a pilot prior to national scale up; antenatal, normal delivery and postnatal services that were initially included as part of the basket of services in a primary care per capita National Health Insurance Scheme (NHIS) provider payment system dropped off the agenda. Under a per capita provider payment system (capitation), accredited health service providers receive in advance, a predetermined lump sum payment to provide a defined package of services for each enrollee with the provider for a fixed period [9, 10]. The predetermined lump is computed to reflect the average cost of providing the defined package of services to the enrolled population. The amount is paid whether or not the enrollees make use of services within the payment period. Providers therefore have strong incentives to minimise their financial cost. Since the compensation package is decided prospectively, providers can maximize the difference between their earnings and costs by simply keeping costs down. There is no limit on the number of times the enrollee can seek services from the provider, and providers therefore have an incentive to limit the quantity of services provided to the patient per visit as a preferred approach to reducing their operating cost [11–13].

In 2010 the National Health Insurance Authority (NHIA) commenced a process to design and implement a per capita provider payment system in Ghana on a pilot basis. The stated objectives for the introduction of the per capita payment system were to: (1) *improve cost containment, efficiency and effectiveness of health services through more rational resource use. (2) share financial risk between schemes, providers and subscribers. (3) introduce managed competition for providers and choice for patients to increase the responsiveness of the health system. (4) correct some imbalances created by using the Ghana Diagnostic Related Groupings payment system for outpatient care such as outpatient supplier-induced demand. (5) simplify claims processing and (6) address difficulties in forecasting and budgeting.* The approach of a pilot in one region before nationwide implementation was to *“enable testing of the overall effectiveness of the designed system in achieving the identified objectives, identify key features of implementation that would be essential for success in scale-up after the pilot”* [11]. Ashanti region where implementation of the capitation was piloted has 19 % of Ghana’s population – making it the region with the largest proportion of Ghana’s population. It reflects the diversity of Ghana from the complex metropolis of Kumasi the regional capital to deprived remote rural areas like parts of the Afram plains [14].

The use of per capita provider payment system in health insurance is not new. Health insurance schemes in middle income countries like Argentina, Brazil, Nicaragua and Thailand have adopted capitation payment as a means to remunerate public and private providers [15]. However, for the lower middle income country in Sub-Saharan Africa that Ghana was in 2010 and currently remains, it was a major innovation. In Ghana, capitation was mentioned in the National Health Insurance law (Act 650) and legislative instrument at the inception of the scheme in 2003 as one of the payment mechanisms to be explored [16, 17] and thus already on the strategic purchasing agenda. However, it remained dormant, largely because of a sense that the experience to implement it was lacking; until it re-emerged in 2010 with the NHIA decision to reform the provider payment system.

Primary care maternity services were included in the capitation basket of services in the initial design, and implementation started in January 2012 in the Ashanti region. However, by March 16, 2012 after less than three months implementation of the policy amid heavy public arena social and political contestation of the policy; primary care maternal health services were removed from the basket of service. The specific research questions this study tries to answer are: Who were the policy actors involved? How did they include and subsequently exclude primary care maternity services in the capitation policy and why? This analysis firstly provides insights on how and why primary care maternal health services got onto the capitation policy agenda, implemented and later removed. Secondly, it contributes to the general understanding of policy agenda setting, formulation and implementation in a LMIC setting.

### National health insurance provider payment mechanisms in Ghana

In September 2003, Ghana passed a National Health Insurance Law (Act 650) to provide the legal backing for the implementation of a national health insurance scheme that would ensure all residents access to basic healthcare services [17]. Implementation started in January 2004. A National Health Insurance Fund (NHIF) established as part of the implementation arrangements had as its funding sources a national health insurance levy of 2.5 % value added tax on selected goods and services, 2.5% of all Social Security and National Insurance Trust (SSNIT) contributions; registration fees from all enrollees and premiums from non SSNIT contributors.

NHIS provider payment mechanisms have evolved over time. In 2004, NHIA started with itemized billing with no standardized fee schedule for services and medicines. Each of the district schemes negotiated with their providers itemized fee rates for services, consumables, and medicines. In the face of growing concerns over inefficiencies such as random price variations for the same procedures and consumables, cumbersome billing and claim vetting procedures and cost escalation, NHIA in 2008, introduced a case based payment mechanism known as the Ghana – Diagnosis Related Groups (G-DRG) for clinical services and procedures; and standardized itemized fees for medicines based on a periodically revised medicine list. The G-DRG is an adaptation of the DRG approach, in that although it has the patient classification system, it does not have cost weights and severity levels. The G-DRG and itemised fees for medicines are applied nationwide for all levels of care from the lowest (Community Health Planning and Services compounds) to the highest (Teaching hospitals), to pay all accredited providers – public, quasi-government, and private – for inpatient and outpatient services. A study of Ghana’s NHIS provider payment and service supply behaviour and incentives by Agyepong et al. (2014) found that financial incentives to service supply were mixed. For example the G-DRG design allows a provider to bill for three visits for outpatient care – the initial visit and two follow-up visits. It could be to the financial advantage of the provider to bill routinely for all three visits regardless of whether the client needed or even made them. On the contrary, the bundled payments of the G-DRG for services were a disincentive to carry out extensive diagnostic investigations whether they were needed or not. Additionally, there was less financial incentive to over prescribe than would be expected under the itemized fee for service billing system, because of the actuality as well as the perception of too low tariffs that negated, in part, incentives to prescribe and dispense unneeded medicines [18].

Payment to providers for services and medicines was and remains retrospective. Section 38 of the legislative instrument (LI 1809) recommended schemes to pay claims within four weeks after receipt from a health care facility. In practice, it takes much longer. Providers file claims, which go through a vetting process in the NHIA district scheme offices or for the higher-level facilities such as teaching and regional hospitals in the computerized central claims processing office of the NHIA before final payment. The claims processes of many provider and district scheme offices remain predominantly manual despite increasing computerization [17, 19].

Maternal health is a national priority and reducing financial barriers is one of governments’ interventions to improve outcomes. Related to this, in 2008 Ghana started implementing its free maternal health care policy under the NHIS and reimburses service providers through the G-DRG payment mechanism for these services. Table 1 describes the benefits under the free maternal care policy [20].

**Table 1:** Benefit under the free maternal care policy

• No premium for fresh registration or renewal of membership
• No processing fee for registration or renewal
• Antenatal period: free antenatal, general services and medicines
• Delivery: free service and medicines, including caesarean
• Postnatal period: free services and medicines
• Full year cover no matter when pregnant woman registers
• Free care for the baby on mother’s NHIS ticket for 90 days
• Alternatively the baby can be treated free on the father or other designated guardian
• After 90 days the child can be registered as an individual under 18 (no premium but processing fee required)

## Methods

### Study design and data collection

We used a case study design because it allows collection and analysis of comprehensive and systematic data at different points in a real-life context to trace policy discussions and change over time [21, 22]. Data was collected between July 2012 and August 2014 using in-depth interviews, document reviews, observations and feedback discussions with respondents. The in-depth interviews were conducted to obtain real-life experiences from key actors involved in the decision making and pilot implementation of the per capita payment system especially in relation to maternity services. The interviews lasting on average 1 h were conducted face to face using a semi-structured guide to investigate how policy actors included and later excluded primary care maternal services from the capitation policy. AK (one of the authors) interviewed twenty-eight respondents summarized in Table 2. For confidentiality, names and positions are not used. Ten of these were identified from the documents review and the rest (18) were suggested by other respondents.

**Table 2:** List of respondents by agency /role in the health sector in relation to capitation

Respondents	Number
Ministry of Health	4
National Health Insurance Authority	4
Ghana Health Service headquarters	2
Ashanti regional health directorate	2
Provider Payment Mechanism Technical Sub Committee	2
Society of Private Medical and Dental Practitioners	2
Christian Health Association of Ghana head office	1
Public health facility in the Ashanti region	4
Christian Health Association of Ghana health facility in Ashanti region	1
Private self-financing (for profit) health facility in Ashanti Region.	3
Government politician	1
Opposition politician	1
Coalition of non-governmental organizations in Health - Ashanti regional representative	1

Document analysis was used to map the sequence of decisions and actions, identify actors’ roles and further triangulate findings with respondent’s information. We conducted content analyses of provider payment mechanism technical subcommittee meeting records and reports (2010–2012); press releases and media discussions from the Ghana News Agency archive related to the policy.

To understand decision making dynamics and interactions in the Ghanaian health sector, a 20 month period of practical attachment at the MOH Policy Planning Monitoring and Evaluation Directorate (PPMED) was undertaken by AK (one of the authors) as a participant observer. The PPMED coordinates policy formulation and strategic planning for the health sector. As a result, there were interactions with the key regional and district health actors during the MOH joint monitoring team visit to Ashanti region (6^th^ - 9^th^ November 2012). Further interactions with key actors during a December 21–22, 2012 national health insurance stakeholder meeting in Accra and a February 12, 2013 capitation evaluation meeting in the Ashanti region gave insights into the varied opinions on the capitation policy.

The initial findings were validated and further substantiated by a presentation for discussion, comments and critique at an August 29, 2014 provider payment mechanism technical subcommittee meeting.

### Analysis concepts

We drew from Mintzberg’s power concept to guide the analysis of what powers policy actors used to control decisions and actions related to the rise and fall of primary care maternal health service capitation policy. Mintzberg (1983) defines power as the capacity to effect (or affect) decisions and actions and labels an actor who seek to control decisions and actions as influencer. Mintzberg argues that influencer’s interpretative manoeuvres ability vary as each tries to use his or her own source of power as means of influence in a politically skilled way. He proposes the sources of power as the control of a resource, a technical skill, or a body of knowledge; authority by virtue of one’s legal and structural position; and access to those who can rely on the other four sources of power [23].

To analyse policy actors’ responses and actions related to the rise and fall of the policy; we drew on the concept of arenas of conflict of Grindle and Thomas (1991). Grindle and Thomas (1991) observed that decisions to change existing practice almost always generate conflict. They described two broad scenarios of reactions or response to policy change – conflict in the public arena and bureaucratic arena. Conflict to policy change in the public arena usually occurs during implementation and when the costs or burden of the reform has a direct impact on the public or on politically important groups in society. On the other hand, conflict in the bureaucratic arena is largely determined by bureaucratic agencies and public official’s response to the change. This usually occurs during policy formulation especially when the administrative content of the policy is high or it is technically complex and requires coordinated efforts of public officials and agencies through consensus building to design the reform [24].

To understand and analyse how providers were able to resist the policy in addition to their use of power; we drew on Sterman’s (2006) concept of policy resistance. Sterman (2006) conceptualises policy resistance as the tendency for a policy to be defeated by a system’s response to the policy itself. He argues policy resistance arises because the system is complex made up of separate but interdependent parts that interact with each other in many ways. The system is therefore dynamic, evolving, interconnected and governed by feedback loops. He further argues that within a system decisions and actions feedback on themselves, triggering others to act thus giving rise to a new situation. Policy actors operate within this complex system and their actions and decisions alter the system and, therefore may trigger unanticipated effects. Others seeking to achieve their goals and acting to restore the balance may also trigger intended and unintended consequences. Policy resistance arises because policy actors are not aware of the full range of feedback surrounding – and created – by their decisions [25].

Drawing upon these concepts, we systematically attempted to reconstruct the case of decisions and actions surrounding the rise and fall of primary care maternal health service capitation policy in the Ashanti region. The information was analysed first to map events and the power sources of key policy actors. A stakeholder analysis of actors as individuals, groups and institutions was done to further understand their position, interest and use of power to influence. Next the evolution of decisions and actions, the formation of groupings were identified. Finally, the analyses were synthesised to reconstruct insofar as possible the case. We acknowledge the difficulty in providing a full explanation of events as they unfolded within the dynamic health system – reconstructing who said what, where, when, to whom and how it was received. To minimise this multiple research methods and data sources were used. Where such data is available, it is noted; otherwise, the gap is noted and possible inferences are made from data analysis.

## Results

### Technical analysis: the rise of primary care maternal health service capitation policy

#### Capitation provider payment: an active policy option

**Fig. 1:**
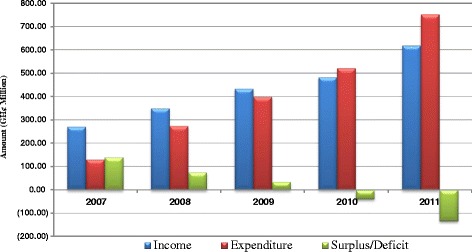
Trend of NHIS income and expenditure 2007 to 2011

Health service cost containment was the main driver for the NHIA provider payment reforms. The financial challenge was twofold – increasing claims cost accompanied by a much lower rate of increase of the NHIF [26, 27] as summarized in Fig. 1 (Trend of NHIS income and expenditure 2007 to 2011) [28]. The NHIA attributed the financial challenge, first to increased number of enrollees. For instance, the number of registered pregnant women more than doubled from 421,234 in 2008 to 1,277,819 in 2010. And, second to overbilling practices such as service providers billing the NHIS for multiple visits that did not occur [20, 29].

#### Primary care maternal health service capitation policy agenda and formulation

The NHIA with the assistance of the World Bank supported health insurance project established the Provider Payment Mechanism Technical Sub-Committee (henceforth Committee), in June 2010. The Committee with health financing and implementation expertise and authority to design the capitation policy comprised officials of the NHIA, MOH, Ghana Health Service (GHS), Christian Health Association of Ghana (CHAG), Korle Bu Teaching Hospital and a national representative of the Society of Private Medical and Dental Practitioners (SPMDP). The Committee assessed the financial situation of the NHIS and noted that the G-DRG payment system had not contained cost particularly outpatient services claims. Furthermore outpatient claims was accounting for 70 % of NHIS claims with an increased average claims of 50 % between 2007 and 2009. To ensure that a routine package of services was paid for by a standard capitation rate across the country, the Committee agreed on a basic basket of service for walk-in outpatient department (OPD) to be paid for by capitation. The original basket of services classified as the primary health care (PHC) bundle is listed in Table 3.

**Table 3:** Capitation basket of services (Primary health care bundle)

1. Primary health care outpatient department consultation
2. Maternity consultation and services with a trained midwife or doctor for antenatal, normal delivery and postnatal
3. Medicines for services included in the capitation package
4. Selected laboratory services that can be performed at all levels, namely:
• Routine Urine
• Malaria Test
• Blood Test
• Pregnancy Test
• Venereal Diseases Research Laboratory Test

The Committee’s technical consideration of the financial and sustainability challenges of the NHIS alongside what should be essential primary health care in Ghana and therefore what should be included in the per capita basket of services presented a window of opportunity to reform not only health financing but also maternal health service delivery. The NHIS per capita provider payment reform was also needed to address some of the long standing challenges associated with delayed reimbursement to providers. There had been numerous instances where providers suspended services to NHIS enrollees because of delayed payments from the NHIA [30, 31] and this may be due to long vetting processes [19] and the fact that NHIA expenditure is higher than its income [32]. The adverse effect of services suspension on maternal health was that expectant mothers had to pay out-of-pocket at the point of use to access a ‘free’ service. The upfront payment to providers that capitation mechanism offers was a potential to reduce if not prevent such happenings. Prompt payments of capitation rate to providers may not be guaranteed, however the delays may be minimal because the amount is predetermined and claims vetting is excluded. Capitation also holds service providers financially responsible for services they deliver and this provides strong incentives for them to integrate activities and reduce cost [33]. Therefore including antenatal, normal delivery and postnatal services which can be provided at primary care level in the capitation basket of service was to ensure continuity in care because capitation payments mechanism could minimise provider’s tendency to suspense services to primary health care.

The Committee also agreed on the following that: (1) a provider must demonstrate the availability of the listed services within the facility to be accredited as a primary care provider to receive a capitation fee. (2) capitation would be limited to this primary health care bundle and all other services would continue to be paid for by the already existing G-DRG for services and itemized fee for medicines [11]. Review of the committee’s reports and meeting records shows decisions including those on per capita rate and enrolment requirement before pilot start were based on consensus building within the Committee; after a back and forth process of discussions. Medicines dropped off the basket of services before it moved into implementation because of stakeholder and technical concerns that it was not clear how best to implement a capitation basket that included medicines in the context of Ghana’s health system. It was decided to continue to pay for all medicines under the itemized fee for service.

#### Primary care maternal health service capitation policy stakeholder education and advocacy

The NHIS is a major health service purchaser and its sustainability is a major national concern. Ways to ensure its sustainability had been discussed at many fora. As a result, at the health sector multi stakeholder November 2011 meeting, participants welcomed the introduction of the capitation policy for primary health care services as a measure to improve efficiency and contain cost [34].

During the pre-implementation phase the Committee and officials of the NHIA held series of fora to inform stakeholders from local to national level on key policy principles. Several of these were covered by the media for example forum with the Asante-Akin South District Assembly at Juaso [35]. District health insurance schemes and the regional implementation committee; representatives of the MOH, GHS, CHAG and health professional bodies; and private self-financing providers were all participants at these fora [36].

The NHIA brought on board politicians to inform and solicit bipartisan support. This included members of the Parliamentary Select Committee on health – elected parliamentarians with the mandate to advice the Parliament on health issues - and members of Parliament representing all constituencies in the Ashanti region. Additionally local government was engaged through the regional coordinating council and the district chief executives in the Ashanti region [36, 37]. However, the political approval from politicians may have influenced resistance from some stakeholders. As a policy implementer noted – *‘involving politicians created the impression that capitation policy was a political issue, putting a political connotation on the policy’* [9/11/2012].

### Contestation: the fall of primary care maternal health service capitation policy

Service providers especially the private self-financing (private for profit) contested and resisted the policy intent and implementation in the run up to and during implementation. Service providers wield a lot of power based on multiple sources including their knowledge, skills, authority, social and professional identity and access to other influencers such as the Minister of Health and NHIS enrollees. The access to NHIS enrollees, the discretion required by the nature of their work, public respect and trust for as well as dependence on their skills and knowledge gave them major “street level bureaucrat” [38] power. The Minister of Health and NHIS enrollees were not actively involved in the policy design, but had the power to influence the policy process when mobilized. These power sources closely parallel those suggested by Mintzberg (1983).

Service providers’ contestation and resistance created unanticipated effects. There was intense media attention and discussions across the country and not only in the Ashanti region where the pilot was taking place. A mobilized pressure group – Ashanti Development Union - sprung up to oppose the capitation policy. Stakeholders called for the policy to be suspended, and private self – financing providers finally suspended services to NHIS subscribers as part of their protest against the policy. Apart from the contestation by the service providers, the fact that 2012 was an election year fueled the public arenas of contestation and high politics as commentators from both sides of the political divide joined the media discussions and increasing acrimony. All these finally cascaded into a crisis situation and gained the attention of the President and the Minister of Health. We discuss in more detail below these stakeholder’s arguments, unanticipated effects, and the committee’s and government’s responses and actions using specific contested issues to illustrate.

#### Stakeholder’s arguments

Policy resistance to including maternity services had started to build up even before implementation finally started in January 2012. The main contested issues included: the per capita rate, the enrolment rate, rationale for using the Ashanti region as a pilot site and ‘all or nothing’ choice scenarios that confronted the service providers.

##### Per capita rate

The per capita rate for the basket of services was computed from a combination of an analysis of historical NHIS annual expenditure on the services in the basket against annual enrolment; and an estimate of the NHIA ability to pay. The per capita rate was then adjusted further for service fixed cost difference between private and public health facilities [11]. The calculation of the per capita rate drew from the G-DRG payment system data. Even in the computation of the G-DRG rates, there had and continued to be challenges related to data quality and completeness and the need therefore to model and estimate. However this was the best data available and the G-DRG system had been developed with it and accepted by providers [18].

Providers – public and private – raised several concerns with the per capita rate and its calculation. First, they felt the per capita rate was too low. In response to this the Committee reassessed and increased the rate by 22 % [36]. However the SPMDP and providers under the Manhyia health insurance scheme disputed and maintained the revised rate was still inadequate. Discrepancies between provider and scheme data on claims and utilization created a data gap and made it difficult for either the NHIA or providers to be certain about the appropriate rate for the PHC bundle.

Providers also argued that the gains made under the free maternal care policy would be derailed under the capitation policy since there would be incentive to reduce service inputs.

‘*Maternity service is a priority for the country and also for the MDGs, maternal mortality will increase if maternity service is put under capitation. Under capitation, there will be restricted services and this will affect the quality of care given - for example the number of antenatal may be reduced by the provider*’ [GHS staff, 9/11/2012].

Thirdly the per capita rate was a single flat rate with no risk adjustment. The data quality problems did not make risk adjustment possible. Providers argued that the type of enrollees played an essential role in the nature of the financial risk borne by providers. They anticipated their risk to be much higher with more enrolled pregnant women.

‘*The outcome of pregnancy is certain and that is delivery. If maternal service is capitated, we (providers) will bear most of the financial risk* [Public Provider, 5/11/2012].

Fourthly and related to the preceding arguments, they anticipated the low rate would ruin their health care business.

‘*This capitation policy will collapse health care system and business in the Ashanti region because the per capita rate is too low and we cannot provides services with such small amount*’ [SPMDP, 7/11/2012].

Finally, providers claimed to be unable to understand the method of rate computation.

*‘We (providers) do not understand how the capitated rate was calculated*’ [SPMDP, 8 /11 /2012].

Table 4 shows the per capita rate for implementation as of July 2011 after the 22 % upward adjustment.

**Table 4:** Per capita rate per health facility ownership for implementation as of July 2011

Provider Ownership	Capitation Rate (GH¢) Clinical Service (Per Member Per Month)	Capitation Rate (GH¢) Medicines (Per Member Per Month)	Total Rate (GH¢) Clinical Service & Medicines (Per Member Per Month)	Total Rate (USD) Clinical Service & Medicines (Per Member Per Month)
Private self-financed	1.11	0.64	1.75	1.16
Government	0.59	0.64	1.23	0.81
Mission-based	0.79	0.64	1.43	0.95

Source: Preferred Primary Provider Group Practice Guidelines, July 2011, National Health Insurance Authority. Conversion from Ghanaian cedis (GH¢) to US dollars; exchange rate at 4.00 pm universal time on 31st July 2011 - 1GH¢ = 0.662USD. http://www.xe.com/currencytables/?from=GHS&date=2011-07-31

##### Enrolment rate

Per capita payment systems use the transfer of an average rate per enrollee. This way in any given period, the money of those who do not use the system helps to take care of those who use the system. Under these circumstances it is essential that 100 % of active enrollees voluntarily chose a preferred primary care provider (PPP) or are administratively assigned to a PPP to avoid short changing providers in the per capita transfers. The method chosen for enrolment to PPP in the Ghana per capita payment system design was voluntary enrolment. It was however acknowledged that it would be impossible to get 100 % voluntary enrolment. The Committee therefore stipulated that an at least 80 % voluntary enrolment rate needed to be attained and then the remaining enrollees would be administratively assigned for the implementation start. But, by December, 31 2011, only 46 % of the active NHIS subscribers had voluntarily enrolled with a PPP. The SPMDP and providers under the Manhyia health insurance scheme contested the start date given that 80 % enrolment to PPP had not been attained. The Committee attributed low enrolment rate to logistics, staffing and financial constraints as well as poor management of the enrolment process. There were also communication challenges such as people considering enrolment information as political propaganda [36]. Because of the slow progress in enrolment, the Committee had already shifted the start date from August 2011 to October 2011 and again to December 2011 in a quest to attain closer to 80 % voluntary enrolment. By October 2011, the Committee felt it was no longer appropriate to keep changing the start date. 2012 was an election year and after the first quarter of the year it would be impossible to introduce any reform as major as capitation. A lot of time, money and effort had already been invested in designing the policy and accompanying programmes and trying to move them into implementation. Moreover, some stakeholders perceived the frequent shift in start date as a sign of weakness and a policy that was doomed to failure.

*‘The continuous changing of the start date did not help, it fuelled the perception that the NHIS is collapsing* ‘[GHS staff, 9/11/2012]. …*‘postponing several times the start date contributed to less confidence in the policy implementation’* [Committee member, 29/08/2014].

The Committee felt that given all these issues, the voluntary target of 80 % should be lowered and other strategies devised to ensure 100 % of the insured had been assigned to a PPP.

##### Suspicions about the rationale for the selection of Ashanti region

The mobilized pressure group – Ashanti development union - and providers questioned the rationale for selecting the region for the pilot. Some claimed the region was chosen because NHIA labelled it a ‘fraud region’.

‘*NHIA brought capitation to the region because they believe there is fraud and abuse here. So the focus is to fight fraud’* [Private self-financing provider, 8/11/2012].

Others claimed the region was punished for its voting patterns. The region is politically described as a New Patriotic Party (NPP) – the party in opposition at the time of introduction of the pilot – ‘stronghold’. The NPP has consistently won the majority parliamentary seats since the start of multi-party democracy in 1992. For instance in the 2008 election, the NPP won 34 parliament seats while the National Democratic Congress (NDC) that won the national presidential election had only three [39]. The timing of the pilot in an election year in addition to placing it in an opposition stronghold also fuelled the suspicions about the intent of the reformers.

‘*Some people believe this is political, this is to punish the region for voting against the government in power. The timing was also wrong, implementing such a policy in an election year in an opposition dominated region, it’s difficult to understand their (NHIA) motive’* [GHS staff, 28/8/2012].

##### ‘All or nothing’ choice scenarios

The policy implementation presented providers with ‘all or nothing’ choice scenarios. Under the capitation policy developed by the Committee any service beyond a normal delivery for example assisted deliveries and caesareans were to be paid for by the G-DRG. Providers who run primary as well as referral care clinics could not opt out of being NHIS provider for primary care under a per capita system and continue to be provider for referral service care.

‘*If you do not participate in capitation, you cannot provide services for NHIS subscribers – unless the subscriber pays out-of-pocket. This is not fair.’* [Private self-financing health facility, 8/11/2012].

Accredited facilities needed to have the capacity to provide the whole basket of services to qualify as a preferred primary care provider. Maternity homes are private facilities run by nurse midwifes. They are licenced purely for the provision of primary maternal care services such as antenatal, delivery, postnatal and family planning. From a legal point of view, Maternity homes could therefore not become preferred primary care providers, since they were not licenced to provide the other components of the primary care per capita package other than antenatal care, normal delivery and postnatal care. Maternity homes accounted for about 12 % of NHIS accredited providers in the region [40, 41]. Maternity homes were particularly concerned about this since though they were only licenced in theory to provide maternity services, many provided other primary care services. To date no one had applied the law strictly, but what would happen under a per capita payment system?

*Within the package there were other services apart from the maternity services, and as such the maternity homes will not be able to be part of the capitation payment system and they were going to lose out’* [NHIA official, 4/10/2012].

‘*The position taken by the NHIA that facilities should provide all the primary care bundle will collapse maternity homes*’ [SPMDP, 7/11/2012].

In response, the Committee recommended maternity homes could be a PPP if they provided evidence of their capacity to provide the whole basket of services. Such evidence included forming a partnership or group practice with another clinic or a community pharmacy shop with written agreements confirming that all the partners understand and had agreed to group together as a ‘primary health care bundle provider’ [41]. This concept of group practice was however new to Ghana. Private providers were unclear how to operationalize it, or even if they wanted to operationalize it. And there were all the other objections to capitation.

#### Unanticipated effects

##### Policy redefined by opposing voices

The private self-financing providers made recommendations to promote their desired outcomes, with contestation starting at the bureaucratic level before implementation start in January 2012. In a petition dated October 5, 2011 to the NHIA chief executive officer, the SPMDP suggested the removal of maternal health service from the basket of services. The SPMDP also recommended new per capita rates between 15–20 times the rates calculated by the NHIA for the PHC bundle as listed in Table 5.

**Table 5:** Per capita rate recommended by the SPMDP in December 2011

Facility	Total Rate (GH¢) Clinical Service & Medicines (Per Member Per Month)	Total Rate (USD) Clinical Service & Medicines (Per Member Per Month)
Hospital	20.57	12.57
Clinic	18.14	11.08
Maternity	9.76	5.96

Source: Recommendation to the NHIA on pilot implementation of capitation in Ashanti region, December 2011, Society of Private Medical and Dental Practitioners

Conversion from Ghanaian cedis (GH¢) to US dollars; exchange rate at 4.00 pm universal time on 31st December 2011 - 1GH¢ = 0.61087USD. http://www.xe.com/currencytables/?from=GHS&date=2011-12-31

The SPMDP based their calculation on the rate of encounter with clients and the existing G-DRG payments as estimated by the society. They noted any amount below their request would immensely affect quality of health care and subsequently collapse private self-financing clinics and maternity homes. According to the SPMDP, increasing cost of general goods and services was making healthcare expensive so primary health care should not be considered as a package of low-cost interventions.

On January 5, 2012 the NHIA therefore organised a meeting to negotiate the basket of service and per capita rate with leaders of the public and private providers, but this ended in chaos with continuing disagreement between the NHIA and the private self-financing providers on the basket of services and the per capita rate. The SPMDP threatened to opt out of capitation. The NHIA also threatened to abrogate all contracts with SPMDP members, stating that private providers that did not implement capitation would not be permitted to provide services under the NHIS [42].

To increase their bargaining power the SPMDP and the Ghana Registered Midwives Association (GRMA) aligned, although they were affected differently by capitation. SPMDP facilities were licensed to provide all the basket of services while Maternity homes were not, despite still providing a broader range of services in practice. SPMDP’s focus was to negotiate a higher per capita rate and GRMA focus was to negotiate approval to provide all the basket of services to NHIS enrollees. To register their objection they moved the discussions to the public arena using the media; and issued a press release on the January 11, 2012 stating: ‘*We shall not start with the pilot capitation under its present form. However, we shall continue to render our services to our Health Insurance Clients using the existing Ghana-Diagnosis Related Groupings (G-DRG) package based on our contract with the National Health Insurance Authority’*. The unfolding resistance and press release did not deter the NHIA’s intent to continue implementation of the per capita payment system despite the strength of private self-financing providers as key health players in the health sector and the Ashanti region. According to the regional health directorate 2010 half year report, the private maternity homes and clinics operated two hundred and seventy-eight (278) out of the five hundred and twenty-seven (527) health facilities (53 %) in the region [43].

##### Increased media attention and calls for policy suspension

The media with its power to instantly reach thousands of people served as a platform for many stakeholders to convey their messages. Intense media discussions built up as stakeholders discussed multiple interpretations of the policy. Discussants, some with inadequate technical understanding shifted to political interpretations that not only contributed to misinform, but also publicized ideas and influenced others [36]. *‘Some politicians and even some officials of the NHIA misunderstood the technical content of the capitation payment system’* [Committee member, 29/08/2014]. For example, a municipal chief executive (a political appointee), stated that the capitation policy was not for ‘*political witch hunting’* but to check corruption as most providers and some NHIS officials had connived to loot the scheme resources [44]. Such statements influenced political discussions and shifted attention away from the intent and purpose of the policy and the technical issues underlying the disagreements.

Multiple interpretations and the unresolved negotiations ultimately fuelled calls for the capitation policy postponement to allow agreement on contested issues. A range of stakeholders - politicians (mainly opposition), private self-financing providers, health professional bodies (Ghana Medical Association and the Pharmaceutical Society of Ghana), and the Ashanti development union joined the call to suspend the pilot [45–48]. The opposition politicians and supporters took to the streets to register their disapproval [49] and the Ashanti development union threatened a demonstration [47].

##### Private providers suspended their services to NHIS enrollees

The NHIA did not postpone the capitation policy and did not give in to the demands of private self-financing providers. Resistance reached its highest point on February 1, 2012, when the private self-financing clinics and Maternity homes operators suspended their services to NHIS enrollees. They stated the policy was imposed by the NHIA to the detriment of quality care and health facilities in the region [50]. The suspension incited further calls to postpone the policy and the opposition parliamentarians perceived it would lead to poor maternal and child health outcomes [51].

### Politics: the governments’ responses and actions

The resulting crisis escalated to a high politics situation, attracting the attention of the President and the Minister of Health. The President in his February 16, 2012 State of the Nation Address to Parliament acknowledged the crisis and called for urgent dialogue and consensus building on NHIS provider payment mechanisms to ensure sustainability [52]. To intervene and build consensus, the Minister of Health met both private and public providers on February 29, 2012. The Minister assured providers of government’s commitment to dialogue with all stakeholders to design a comprehensive and sustainable health financing policy for the benefit of all. He reiterated government would not impose any policy to the detriment of any group of people and pledged to convey their issues to the President for immediate action [48].

Parliamentary and presidential elections were due to be held in December 2012. Government stood to gain political points by listening and responding favourably to the opposing voices and to lose if they did not. By March 16, 2012, the government had taken a decision to have primary care maternal health service removed from the basket of service. NHIA was to reimburse accredited health facilities through the G-DRG payment mechanism [53].

It is of interest to note that four years on, in 2016, capitation is being scaled up in a step wise fashion with three regions of Ghana set to begin implementation any time soon. The process has been quiet and relatively free of the rancour and contestation of the original pilot to date. The reasons are a story in their own right. However part of it is definitely the organizational learning that occurred from the experiences of the pilot.

## Discussion

This case illustrates the tensions and complicated relationships between technical consideration, contestation and political responsiveness in policy processes that combine to determine the outcomes of policy agenda setting and formulation, with the result of implementation processes sometimes leading backwards to a revision of the policy agenda and formulation.

The capitation payment mechanism was already legitimised by Law (Act 650) but dormant until multiple concern about financial challenges of running NHIS, high outpatient (primary health care) claims and the increased experience and technical skills with provider payment in the country made it just ‘right’ to implement primary care maternal health care services. We reason with Cook and Skogan (1990) that factors such as policy legitimisation, multiple source concern of an issue and a ‘ripe climate’ contribute to elevate the issue onto the agenda for implementation [54].

Actions and decisions of opposing stakeholders (policy influencers) led to the fall of primary care maternal health services from the capitation policy. Opposing stakeholders in this case, relied on their professional, political and social sources of power to convince those to whom they had access, project their problems and by that reframed the issues to their benefit. They created a system of meaning [55] as they reframed issues from their understanding as well as ideas from others to manipulate revision of the policy. As suggested by Stone (2012 p. 176) policy actors use interpretation as strategic manipulation tool to frame issues to lend legitimacy and attract support to a course of action [56]. In our case and also noted by Agyei-Baffour et al. (2013) [57], the media was venue for information and rebuttal; and a breeding ground for multiple policy frames as stakeholders convey their ideas and influenced others. Decision making process related to the policy moved beyond the bureaucracy of the NHIA into the public arena as media discussants and private self-financing providers reframed the policy issues. In the bureaucratic arena, though there were technical disagreements on what to include in the policy design, decisions were based on consensus. Conversely, in the public arena discussion, decisions were based on the media discussants and private self-financing providers’ ability to manipulate interpretations of the policy in a politically skilful way to their benefit [23] than facts. They used frames such as – “derail maternal health”; “political punishment”; “fraud region”; and “collapse health care” to make their arguments and gain political attention.

Within the public arena, not only did opposing stakeholders leverage their professional, political and social power to reframe issues, but also benefited from the context within which the policy was implemented to justify their actions. For instance, the Ashanti development group based on the political context – a ‘stronghold’ of NPP - questioned the rationale for introducing the policy in the region. Also, the private providers gained a high bargaining power and were able to resist the policy because they operated about 53 % of the health facilities in the region [43]. The fact is that private providers’ resistance imposed limits on the NHIA and the Committee’s power on the policy implementation.

Limits on power by resistance contribute to the outcomes of power relations [58] and the outcomes at different stages of the policy process. In this case, power relations existed between the NHIA and Committee on one side and the private providers. During the formulation process, the NHIA and Committee with the authority and capacity and upper hand within the bureaucratic arena designed the capitation policy for implementation. On the other hand during the implementation process there was a shift in power; private providers with professional knowledge and skill to implement policies benefited from all the policy contestation within the public arena gained the upper hand and in effect influenced the removal of maternal primary care from the capitation policy.

Despite the recognition of the importance of stakeholder engagement and the use of a multi-stakeholder Committee that included providers; stakeholder identification, analysis and consultation was perhaps inadequate. Contestation is often an inevitable part of policy reform, and reform as major as provider payment with all the incentives inherent in different payment methods holds huge potential for contestation. More careful stakeholder analysis as part of the design and implementation process might have perhaps made some of the problems that precipitated a crisis e.g. selecting an opposition region for pilot of major reform; anticipated and perhaps avoided.

So what started as a seemingly quiet negotiation between the NHIA and private providers resulted in a dispute; and like a ‘snowball’ lead to a series of unanticipated effects. And as noted by Sterman (2006) and others, these unanticipated effects are spontaneous and difficult to predict, and feeds back on its self - creating new situations [25, 59]. In this case, the new situation – multiple issue reframing - intensified the attention and interest of the President and Minister of Health in the policy. Because, politics is driven by how people interpret and reframe information; and as such political actors strive to control interpretations [56] and debunk any unfavourable ones.

So, the policy with the opportunity to contain NHIA cost and improve continuous access to maternal health care was overturn by high politics and political responsiveness of the government. In a nutshell, a strong competing voice emerged within an enabling environment to dispute the policy through repeated multiple issue reframes. These factors are similar to those proposed by Cook and Skogan (1990) in their work on the fall of criminal victimization of the elderly from government’s agenda [54].

## Conclusion

Policy formulation and implementation therefore is not only about technical considerations but also how policy influencers’ particularly opposing actors frame and reframe issues to generate political attention and response. The tensions and complicated relationships between technical consideration, contestation and political responsiveness in the capitation policy processes raises some questions we pose for policy dialogue and further research. What is the relationship between government policy makers and private service providers in terms of government policy implementation? How is health care service cost determined? The dynamics of this relationship and how health care service delivery cost is calculated are vital for the overall health care system quest to attain universal health coverage and critical for government interventions to improve access to health care services especially in areas dominantly serviced by private providers.

The tensions and complicated relationships between technical considerations and politics and bureaucratic versus public arenas of conflict are important influences that can cause items to rise and fall on policy agendas.

## Abbreviations

CHAG, Christian Health Association of Ghana; GHS, Ghana Health Service; GMA, Ghana Medical Association; GRMA, Ghana Registered Midwives Association; LI, Legislative Instrument; LMIC, Low and Middle Income Country; MDG, Millennium Development Goals; MOH, Ministry of Health; NDC, National Democratic Congress; NHIA, National Health Insurance Authority; NHIF, National Health Insurance Fund; NHIS, National Health Insurance Scheme; NPP, National Patriotic Party; PPME, Policy Planning Monitoring and Evaluation; PPMTSC, Provider Payment Mechanism Technical Sub Committee; PPP, Preferred Primary Provider; PSGH, Pharmaceutical Society of Ghana; SPMDP, Society of Private Medical and Dental Practitioners; SSNIT, Social Security and National Insurance Trust
